# Conjunctival *FOXP*_3_ in Trachoma: T Cells Not Specified

**DOI:** 10.1371/journal.pmed.0030506

**Published:** 2006-11-28

**Authors:** Michael Probst-Kepper

**Affiliations:** Hannover Medical School, Hannover, Germany

The article by Faal et al. [1] describes differences in the level of *FOXP* *_3_* mRNA expression in the conjunctiva of patients with Chlamydia trachomatis infection. The authors suggest that their results may indicate a role for regulatory T cells in the resolution of conjunctival immune response, since *FOXP* *_3_* mRNA remained elevated even if clinical disease signs were present in the absence of infection.

But suggestions about the function of regulatory T cells in diseases based only on the quantification of *FOXP* *_3_* mRNA should be considered with great caution. According to the current state-of-the-art it is well established that *FOXP* *_3_* does not specify human regulatory T cells since it is expressed at the mRNA and the protein levels to different extents protein at [2–5]. Thus, without quantification of FOXP_3_ the single cell level in conjunction with reliable markers of human regulatory T cells, e.g., the recently identified lack of *CD127* expression on regulatory T cells [6,7], the potential contribution or association of regulatory T cells with a disease cannot be assessed.
